# Cognitive P300 potential in subjects with diabetes mellitus

**DOI:** 10.1016/S1808-8694(15)31311-2

**Published:** 2015-10-20

**Authors:** Kátia de Freitas Alvarenga, Josilene Luciene Duarte, Daniela Polo Camargo da Silva, Raquel Sampaio Agostinho-Pesse, Carlos Antonio Negrato, Orozimbo Alves Costa

**Affiliations:** ^1^ Speech and hearing therapist – Ph.D., Professor, Course of Speech and Hearing Therapy, FOB/USP; ^2^ Speech and hearing therapist - Specialized in Audiology, FOB/USP; ^3^ Speech and hearing therapist – Improvement course under way, Medical School, Unesp Botucatu; ^4^ Speech and hearing therapist - Master studies under course in Pediatrics, Medical School, Unesp Botucatu; ^5^ Endocrinologist, Ph.D. studies under course in Obstetric Gynecology, Medical School, Unesp Botucatu; ^6^ Otorhinolaryngologist - Full Professor, Course of Speech and Hearing Therapy, FOB-USP, vice-coordinator, CPA, HRAC-USP

**Keywords:** diabetes mellitus, hearing loss, event-related potentials, p300, audiometry

## Abstract

Diabetes Mellitus may lead to alterations in the eyes, kidneys, cranial nerves, peripheral nerves, ears etc. The cognitive function also seems to be compromised in subjects presented with Diabetes Mellitus, since the cortical and subcortical structures responsible for this function are hindered in some insulin-dependent patients. The cognitive potential P300 has been used as an objective procedure to assess cerebral cognitive functions.

**Aim:**

To analyze the sensitivity of P300 cognitive potential for the detection of alterations on the auditory cortex secondary to Diabetes Mellitus.

**Study design:**

transversal cohort.

**Material and Method:**

Sixteen diabetic subjects of both genders aged 7 to 71 years, and seventeen non-diabetic individuals at the same age range participated in this study. The evaluation procedures were pure tone audiometry (PTA) and P300 cognitive potential. Glycemia of the group presented with Diabetes was assessed prior to applying P300.

**Results:**

No statistically significant difference was shown for PTA results. A statically significant difference was observed between groups when analyzing the latency of P300 component measured in Fz. There was a correlation between glycemia and latency and amplitude of P300.

**Conclusion:**

The investigation of the cognitive potential of P300 is an important procedure for prevention and early diagnosis of neurological changes in individuals presented with Diabetes Mellitus.

## INTRODUCTION

Diabetes mellitus is a chronic condition whose onset is given when the pancreas does not produce enough amount of insulin or when the body does not manage to effectively use the produced insulin. Abnormalities to production and/or action of insulin may take to hyperglycemia. Prevalence of diabetes varies a lot in all different regions of the world and within the same regions, and we expect that by 2025, the population of diabetic patients in the world will double when compared to the number of existing diabetic patients in 2000 (150 million to 300 million). Diabetes Mellitus is the most prevalent chronic-degenerative disease in our days.

There are four main types of diabetes: 1) type 1 - there is autoimmune destruction of pancreas beta cells that lead to inability to produce insulin, requiring insulin injections to ensure survival of patients; 2) type 2 - it is characterized by affections to action and production of insulin and there may be predominance of one situation over the other, and normally both of them are present. Metabolic control is normally obtained with diet, physical exercises and/or concomitant use of oral hypoglycemic agents, and insulin may be used for treatment; 3) secondary diabetes - type of diabetes in which the causal factor is known, such as some genetic defects that result in abnormalities of beta cell function or insulin action, some pancreatic and endocrine diseases, or those caused by some drugs; 4) gestational diabetes - it is primarily diagnosed during pregnancy and it may disappear at the end of the pregnancy period.

In chronic complications of Diabetes Mellitus there may be presence of eye, kidney, cranial nerves, peripheral nerves and ear affections. In the hearing system, there may be atrophy of spiral ganglion, degeneration of myelin sheath of the eighth cranial nerve, reduction of the number of nervous fibers on spiral lamina, or thickness of capillary wall of vascular stria and of small arteries inside the auditory canal^1^. In histology, inner ear affections are found in 50% of the people with diagnosis of Diabetes Mellitus^2^.

The hearing loss detected in subjects with diabetes mellitus is characterized as bilateral, symmetrical sensorineural loss more marked in high frequencies^3^, ^4^. However, it has been observed that in diabetes type 1, the occurrence of hearing loss is associated with age, duration of disease and presence of neuropathy^5^, ^6^.

Cognitive function may also seem to be affected in subjects with diabetes mellitus, given that cortical and subcortical structures responsible for this function are altered in some insulin-dependent patients^7^.

Cognitive P300 potential has been used as an objective procedures to assess cerebral cognitive function, and it seems to be the right tool to check sequelae caused by hypoglycemia in the hippocampus region^8^, ^9^. In general, we can observe increased latency of cognitive potential P300 both in subjects with diabetes mellitus types 1 and 2^10^, ^11^, and in diabetes type 2 this fact may take place when there are no clinical signs of nervous system damage^12^. P300 cognitive potential is discussed as the most sensitive potential to detect subclinical effects of acute hypoglycemia, when compared to psychometric tests and electroencephalogram^13^.

Studies demonstrated significant correlation between P300 cognitive potential latency and duration of diabetes mellitus, and even though they did not observe it when analyzing the number of episodes of hypoglycemic coma. It indicates that in the evolution of glycemia impairment, long term metabolic abnormalities are more relevant than isolated episodes of hypoglycemia^14^. Prolonged hypoglycemia may cause permanent damage to the brain, especially in insulin-dependent diabetic children^15^.

Thus, the purpose of the present study was to analyze sensitivity of P300 potential to detect cognitive function affections in subjects with diabetes mellitus.^16^

## MATERIAL AND METHOD

The present study was conducted at Audiological Research Center (CPA), Hospital de Reabilitaçã o de Anomalias Craniofaciais e Clínica de Fonoaudiologia, Dental Science School, Bauru, University of Sao Paulo, campus Bauru – SP.

### Selection of Sample

Subjects were referred by the endocrinologist of Diabetic Association of Bauru/Sao Paulo. The inclusion criterion was previous diagnosis of diabetes mellitus. The control group was matched by gender, age and grade of hearing loss to exclude these variables from the exam analysis. In both groups, we excluded the following cases:

• history of other risk factors for hearing loss and/or conventional assessment that presented conductive hearing loss, confirmed by acoustic immittance measurement;
• presence of non-auditory associated disorders that could lead to long-latency potentials, such as neurological diseases or syndromes.

We did not exclude subjects with hearing loss, given that auditory evoked event-related potentials suffer more influence of factors such as motivation of subjects to conduct the test, attention to stimuli, and level of difference between presented stimuli.

### Material

The study comprised 33 subjects and 16 had diagnosis of diabetes mellitus, both genders and ages ranging from 7 to 71 years (diabetic group) and 17 non-diabetic patients matched by gender, age and hearing loss (control group). All subjects received a letter of information and signed the informed consent term to participate in the study.

### Process of Assessment

#### Pure tone audiometry

Pure tone audiometry (PTA) was conducted in soundproof booth, audiometer Madsen, model Midmate 622, earphones TDH-39, calibrated according to ANSI-69. We investigated pure tone thresholds in frequencies 0.5 to 8 kHz by air conduction, being considered normal ≤ 25 dB HL.

#### Cognitive P300 Potential

Auditory evoked potentials refer to changes in electrical activity that occur in the peripheral and central auditory systems in view of an acoustic or electrical stimulus. Evoked potential of the brain is a response to an internal event, such as perception or cognition, and it is named event-related potential, also considered an endogenous potential. In this category, we can include cognitive P300 potential.

Cognitive P300 potential occurs in subjects that consciously recognize the presence of an acoustic stimulus presented by tone burst or speech. Thus, to record P300, we use oddball paradigm, which is characterized by random presentations of stimulus, considered as rare during the presentation of another frequent stimulus. In case of the use of tone burst, stimuli differ concerning frequency, intensity and duration.

In this study, the investigation of P300 cognitive potential was performed in a silent room using insertion phones 3A. We used disposal electrodes for ECG AG/AGCL with gel and clamp type wires to allow the use of this electrode. To start electrophysiological assessment, it was necessary to have electrodes with individual impedance below 5KΩ and impedance between them below 2KΩ. The test was conducted with subjects lying down comfortably and with closed eyes (elimination of artifact caused by ocular movement). Active electrodes were placed in Cz and Fz and connected to input 1 of channels 1 and 2, respectively, of pre-amplifier. Reference electrodes were placed on right and left mastoid and connected to input 2 of channels 1 and 2 of the pre-amplifiers and the ground electrode was place on position Fpz.

We used tone burst stimuli in frequency of 2kHz for rare stimulus, presented randomly in likelihood of 20%, mixed with frequent tone burst of 1kHz, presenting likelihood of 80%, with 25ms rise and 50ms plateau, moderate intensity of 70dB, speed of 1 stimulus every second and use of pass filter 1 to 125Hz. Initial registration was filtered with low-pass digital filter for cut-off frequency of 25Hz. The subjects were asked to identify the rare stimulus, counting in loud voice.

To search for the cognitive potential P300 we used the device Biologic's Evoked Potential System (EP).

Glucose levels were measured in all diabetic patients after placement of electrodes and insertion phones before P300. To that end, we used device Advantage, Accu-check Product, Roche, with Advantage, glucose tape.

#### Parameters of results' analyses

Out of the total data collected, the important figures were pure tone thresholds for right and left ears measured by air conduction in frequencies of 0.5 to 8kHz and absolute latency of components N2 and P300 and amplitude (amp) of P300, recorded in Fz and Cz. We considered as presence of P300 wave when it was simultaneously recorded in Fz and Cz.

#### Statistical analysis

The results of pure tone audiometry were analyzed by descriptive analysis to calculate mean, standard deviation, χ^2^ test to compare control and diabetic groups, analysis of variance with repetitive measurements considering both right and left sides and control and diabetic groups. Results of P300 potential were also analyzed with descriptive analysis to calculate mean and standard deviation, Student t test to compare absolute latency of components N2 and P300 and amplitude, measured in Cz and Fz, for control group and diabetics, and Pearson correlation to check correlation between glucose level and absolute latency of components N2 and P300 and P300 amplitude, measured in Cz and Fz, respectively.

## RESULTS

In Table 1, we show occurrence of hearing loss seen in pure tone audiometry and the results of χ^2^ test comparing control and diabetic groups, showing that there were no statistically significant differences between them.

**Table 1:** Distribution of subjects in the control and diabetic groups according to presence or absence of hearing loss detected with pure tone audiometry. Result of χ^2^ test comparing control and diabetic patients.

PURE TONE AUDIOMETRY				
	NORMAL		HEARING LOSS	
	n	%	n	%
Control	6	35	11	65
Diabetic	5	31	11	69
TOTAL	11	33	22	67
χ^2^	0.881			

*p < 0.005 – statistically significant difference.

In table 2, we show the results of the statistical analysis to compare control and diabetic groups using Variance Analysis in repetitive measurements, whose factors were tested and analyzed in groups, plus auditory thresholds obtained in PTA for each tested frequency.

**Table 2:** Statistical Analysis to compare control and diabetic groups using Variance Analysis (repetitive measures with repetition factor side and repetition factor group) concerning pure tone thresholds for each tested frequency.

	0.5	1	2	3	4	6	8	KHz
Control x								
Diabetic (p)	0.212	0.201	0.262	0.235	0.499	0.424	0.604	

p ≤ 0.05 – statistically significant difference.

Distribution of subjects in the control and diabetic groups, according to level of hearing loss detected in pure tone audiometry, considering means of auditory thresholds in 0.5, 1 and 2 kHz are found in Table 3. In the same table, we found results of χ^2^ test by comparing control and diabetic groups.

**Table 3:** Distribution of subjects in control and diabetic groups according to presence or absence of hearing loss detected in pure tone audiometry considering mean of auditory thresholds in 0.5, 1 and 2 kHz. Result of the test χ^2^ comparing control and diabetic group.

PURE TONE AUDIOMETRY – hearing loss
Control x Diabetic (p) 0,1

p ≤ 0.05 – statistically significant difference.

Table 4 presents descriptive statistical analysis of P300 and absolute latencies of components N2 and P300, in ms, and amplitude of P300 recorded in Fz and Cz.

**Table 4:** Descriptive statistical analysis of P300 cognitive potential concerning absolute latency of components N2 and P300, in ms, and amplitude of P300 recorded in Fz and Cz.

P300 COGNITIVE POTENTIAL				
	CONTROL		DIABETIC	
	X	SD	X	SD
CzN2	229.31	14.61	232.38	28.55
CzP300	324.55	37.47	355.07	41.31
CzP300-amp	2.84	2.99	1.91	1.08
FzN2	232.49	12.7	234.40	31.79
FzP300	333.55	26.28	350.95	41.15
FzP300-amp	3.27	4.35	1.98	0.85

Table 5 presents results of the statistical analysis comparing control and diabetic groups using t Student test and absolute latency of components N2 and P300 in ms and amplitude of P300 recorded in Fz and Cz, respectively.

**Table 5:** Statistical analysis to compare control and diabetic groups with t Student test concerning absolute latency of components N2 and P300, in ms, and amplitude of P300 recorded in Fz and Cz, respectively.

P300 COGNITIVE POTENTIAL						
	FzN2	FzP300	FzP300 amp	CzN2	CzP300	CzP300 amp
Control x Diabetic	0.82	0.15	0.25	0.69	0.03 *	0.25

^*^ * p < 0.05 – statistically significant difference.

The correlations between glucose level collected before the study of P300 and components N2Cz, N2Fz, P300Cz and P300Fz, P300Cz amp, P300Fz amp, using Pearson correlation test are described in Table 6.

**Table 6:** Correlation between glucose level collected in diabetic groups before the study of P300 cognitive potential and components N2Cz, N2Fz, P300Cz and P300Fz, P300Cz amp, P300Fz amp, using Pearson correlation test.

STATISTICAL ANALYSIS –Pearson Correlation						
	FzN2	FzP300	FzP300 amp	CzN2	CzP300	CzP300 amp
*p*	0.027 *	0.141	0.009 *	0.083	0.043 *	0.283

^*^ * p < 0.05 – statistically significant difference.

**Graph 1:**
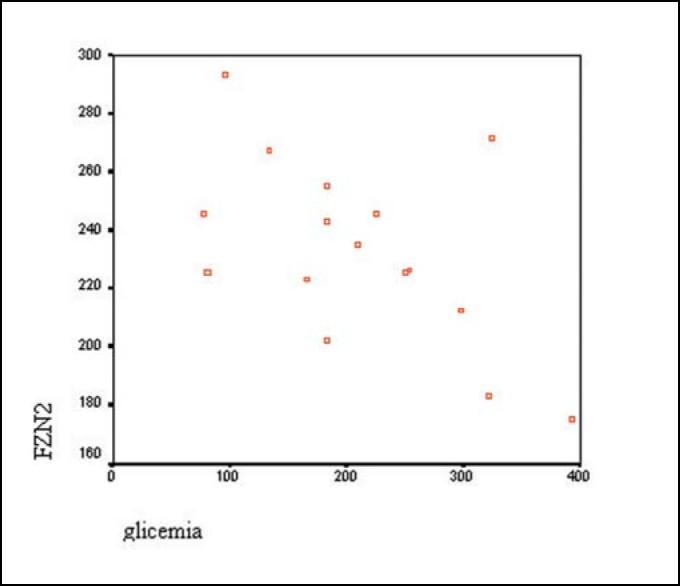
Correlation between latency FZN2 (ms) and glucose level (mg/dl).

**Graph 2:**
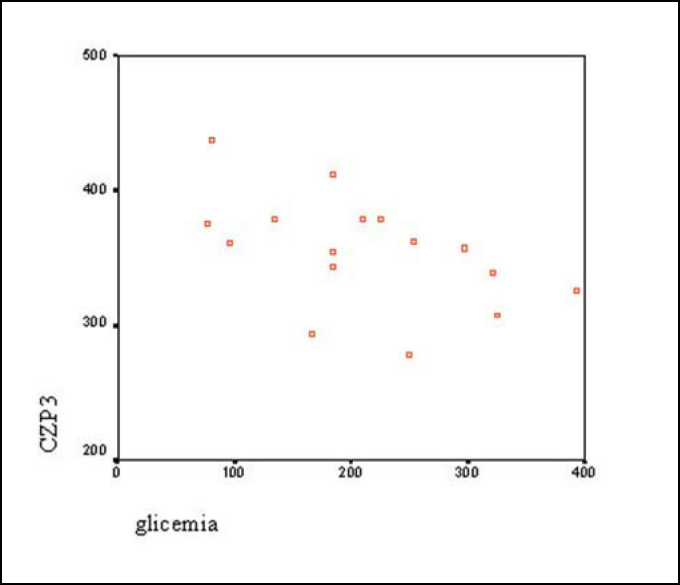
Correlation between latency of CZP3 (ms) and glucose level (mg/dl).

**Graph 3:**
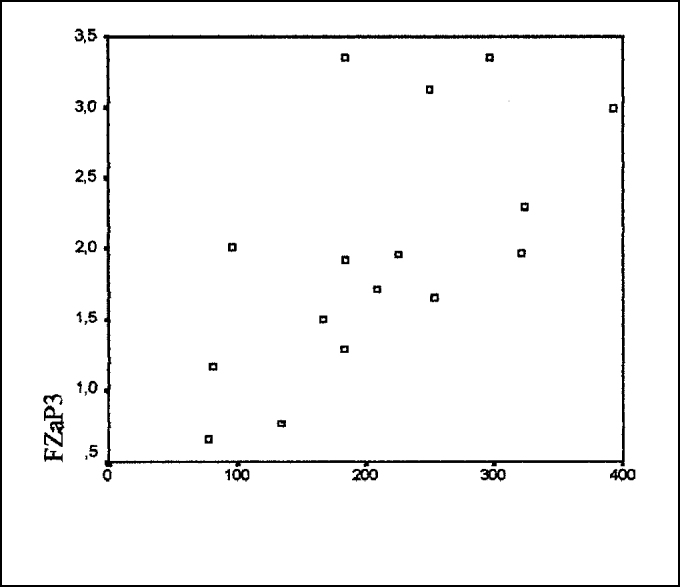
Correlation between amplitude of FZP300 (¼v) and glucose level (mg/dl).

Graphs 1, 2 and 3 show the correlation between component FzP300, considering latency and amplitude of glucose level, respectively.

## DISCUSSION

The fact that there were no statistically significant differences between control and diabetic groups concerning occurrence of hearing loss (Table 1) may be justified by age range of the studied group - seven to 72 years in the control group and seven to 71 years in the diabetic group, counting on the influence of aging in both groups^1^. It is important to point out that all subjects with hearing loss, both in the control and diabetic groups, presented age older than 35 years.

The comparative analysis of hearing thresholds obtained for each tested frequency in pure tone audiometry did not show statistically significant differences between the groups (Table 2). It is was possible to detect similarity in the configuration of audiometric curves in control and diabetic patients, and low sounds had auditory thresholds more preserved than high frequencies, characterizing descending audiometric curve in both groups. This type of configuration is common both in auditory loss resulting from aging and in diabetes mellitus, owing to initial affection to the cochlea base, region responsible for high frequencies. Our findings are in accordance with previous studies^2^, ^3^, ^4^, ^5^. Similar results were observed when we considered the classification of hearing loss according to mean frequencies of 0.5, 1 and 2 kHz. Our findings are in accordance with the results of previous studies^4^, ^5^.

As to P300 cognitive potential, we recorded components N2 and P300 in all subjects of the control group and diabetic group.

Upon investigating P300, we observed increased latency of P300 in Cz, presenting value of 324.55 ± 37.47 ms in the control group and 355.07± 41.31 ms in the diabetic group, with statistically significant difference (Tables 4 and 5). We did not detect statistically significant difference between groups when we analyzed N2 in Cz and Fz. These results suggest increase in latency of P300 in diabetic patients, reflecting possible affections to attention process, auditory discrimination, memory and semantic perspective of these subjects. In the literature, some studies with auditory evoked potentials demonstrated affection to P300 in diabetic patients^6^, ^7^; however, others did not find the same abnormality^8^.

According to Pearson correlation test, there was statistically significant correlation between latency of components FZN2 (p = 0.027) and CZP3 (p = 0.043) and amplitude of component FZP3 amp (p = 0.009) with level of blood glucose. However, we did not observe the same component FZP3 (p = 0.141), CZN2 (p = 0.083) and CZP3 amp (p = 0.283) (Table 6 and Graphs 1, 2 and 3).

Results demonstrated that reduction of glucose level in diabetic subjects takes to increase in latency and reduction of amplitude of component P300, suggesting that there is central auditory system dysfunction. Considering that nervous tissue is glucose-dependent, that is, it depends on stable glucose levels in ideal situations, episodes of hypoglycemia for prolonged periods of time may take the subject to significant neurological affections^9^.

Thus, the investigation of P300 cognitive potential may be an important procedure for prevention and early diagnosis of neurological affections in subjects with Diabetes Mellitus^10^, ^11^.

It is important to highlight that normally in these studies conducted with diabetes type 1 patients, differently from the present study, in which most of the population was comprised by type 2 diabetes patients, we demonstrated that hearing affection may occur in both types of diabetes mellitus.

## CONCLUSION

The results obtained with the present study allowed the conclusion that P300 cognitive potential investigation is an important procedure to prevent and early diagnose neurological affections in subjects with Diabetes Mellitus types 1 and 2.

## ACKNOWLEDGMENT

Study financed by Fundaçã o de Amparo a Pesquisa do Estado de São Paulo (FAPESP).
